# Macular function in preterm children at school age

**DOI:** 10.1007/s10633-016-9564-8

**Published:** 2016-11-12

**Authors:** Hanna Åkerblom, Sten Andreasson, Gerd Holmström

**Affiliations:** 1 Department of Neuroscience/Ophthalmology, Uppsala University, 751 24 Uppsala, Sweden; 2 Department of Ophthalmology, Lund University Hospital, 221 85 Lund, Sweden

**Keywords:** Macula, Multifocal electroretinogram (mfERG), Optical coherent tomography (OCT), Preterm children

## Abstract

**Purpose:**

Macular development is a complex process that starts by mid-gestation and continues several years after birth. A preterm birth could affect this development, causing increased thickness in the central macula, but the effect of the macular function remains uncertain. The aim of this study was to investigate the macular function measured with multifocal electroretinography (mfERG), in former preterm children and compare with healthy controls. A second aim was to correlate central macular function with central macular thickness measured with optical coherent tomography (OCT), in the preterm group.

**Methods:**

Fifteen former preterm children born before 32 weeks of gestation were included in the study. MfERG results from 12 children acted as controls. Visual acuity, refraction in cycloplegia and mfERG were carried out in all children, and optical coherent tomography (OCT) was performed in the preterm children. Main outcomes were P1 amplitudes and implicit times for Rings 1–5 and “sum of groups” of the mfERG, and central macula thickness in area A1 measured with OCT.

**Results:**

The P1 amplitudes were reduced in Rings 1–5 and “Sum of groups” in the preterm children compared to controls. There were no significant correlation between P1 amplitude or implicit times in Ring 1 and central macular thickness in the preterm group.

**Conclusions:**

Macular function is reduced in former preterm children compared to children born at term. This suggests that the structural changes with a thicker central retina can have an effect on function and may be one, of probably several, explanations for visual dysfunction in preterm children at school age.

## Introduction

The macular region is the last part of the retina to develop, and the development is not completed until several years after birth [1]. However, major processes in macular development take place during the second half of pregnancy. Histological studies show how the ganglion cells and inner retinal cells migrate peripherally, and the cone photoreceptors migrate centrally, elongate and become packed tightly together to form the foveal dimple [2]. More recent studies with hand-held optical coherent tomography (OCT) performed in children born preterm and at term have confirmed these developmental processes [3–5].

Normal development of the macular region seems to be disturbed in children born prematurely. When measured with optical coherent tomography (OCT), the innermost area of the macula is thicker in children born preterm compared to children born at term and children with the lowest gestational age have the thickest central maculae [6]. Further, the visual functions of preterm children are affected at school age, including reduced visual acuity and affected contrast vision [7, 8]. In some children, this can be explained by neonatal complications such as retinopathy of prematurity (ROP) and neurological complications such as intraventricular haemorrhages and periventricular leukomalacia, However, even in the absence of ROP and evident neurological complications, visual functions can still be affected and the reason for this is not clear [9].

The aim of this study was to evaluate central macular function with multifocal electroretinography (mfERG) in former preterm children and compare the findings with those of children born at term. A second aim was to correlate central macular function with central macular thickness measured with OCT, in the preterm group.

## Materials and methods

Former preterm children born before 32 weeks of gestation were asked together with their caregivers, to participate in this study, and 15 children gave their informed consent. During the neonatal period, the children were screened for ROP at Uppsala University Hospital. Six children had no ROP detected at screening, seven had mild ROP (stage 1–2), and two had severe ROP (stage 3) and were treated previously with laser photocoagulation.

MfERG results from 12 healthy children between 8 and 19 years of age, with normal visual acuity, acted as controls.

Descriptive data of the children in the preterm group are presented in Table 1.

**Table 1:** Descriptive data for the preterm group, with and without ROP

	Age (years) Median (range)	Gender ♀/♂	GA (weeks) Median (range)	BW (g) Median (range)	VA (Snellen) Median (range)	Spherical equivalent Median (range)
Total *n* = 15	13 (9–17)	11/4	30.0 (24–32)	1231 (611–2030)	1.0 (0.6–1.3)	+0.63 (−1.5–+2.38)
No ROP *n* = 6	14.0 (10–17)	5/1	31.0 (29–32)	1700 (1231–2030)	1.0 (0.6–1.25)	+0.31 (−1.5–+0.88)
ROP *n* = 9	13.0 (9–14)	6/3	27.5 (24–30)	901 (611–1614)	1.0 (1.0–1.3)	+0.81 (0.0–+2.38)

Visual acuity and spherical equivalent in right eyes

*GA* gestational age at birth, *BW* birth weight, *VA* visual acuity

The mfERG examinations took place at the Department of Ophthalmology at Lund University Hospital and were performed by two experienced technicians.

Best-corrected visual acuity was assessed, and the pupils were dilated with topical 1% cyclopentolate. MfERG was recorded using the visual evoked response imaging system (VERIS 6 EDI, San Mateo, CA, USA). After applying topical anaesthesia in the eye, a Burian-Allen bipolar contact lens was applied on the cornea of one eye and the other eye was covered with an eye patch. A ground electrode was placed on the forehead. A protocol with 103 black and white hexagonal elements displayed in a cathode ray tube (CRT) monitor was used. Hexagons were scaled to eccentricity and were altered in a pseudorandomized sequence at 75 Hz frame rate, and ERG responses were recorded in periods of 30 s, 16 times for each eye. The stimulation parameters were in accordance with recommendations of the ISCEV [10]. Fixation was continually monitored with a camera using infrared light at the edge of the corneal electrode to illuminate the fundus.

**Fig. 1:**
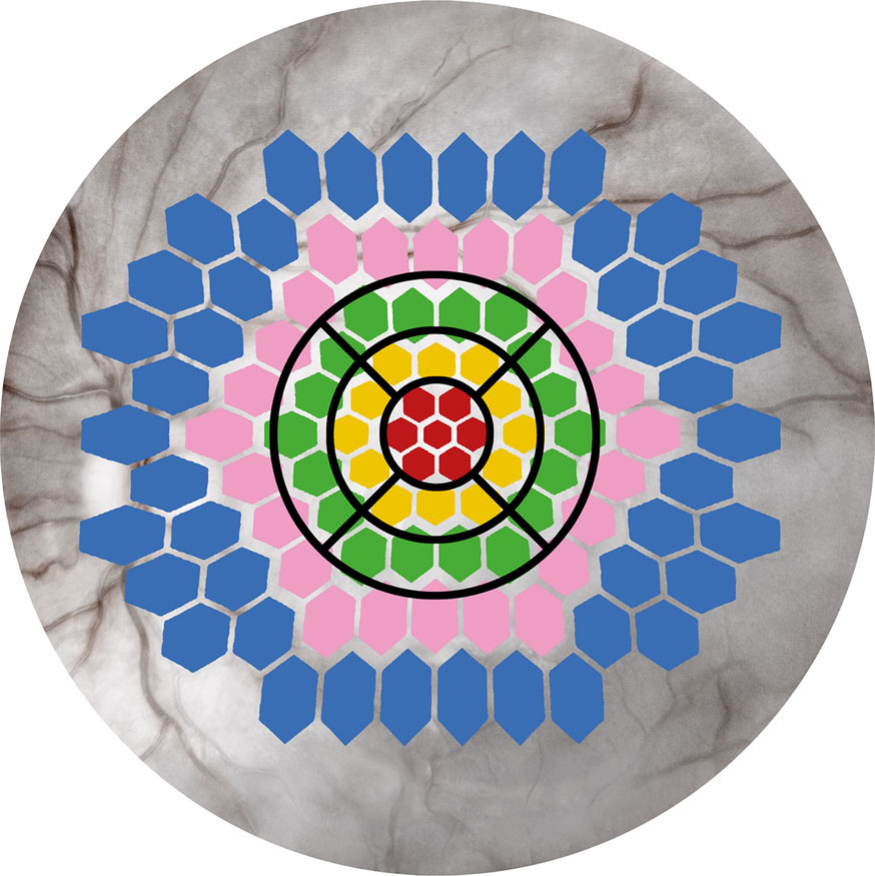
Schematic drawing of the 103 hexagons of the multifocal ERG, with Rings 1–5 marked with *different colours*, and the ETDRS map of the optical coherent tomography (OCT)

Results from right eyes in the preterm group and right or left eyes in the control group were used. The amplitudes and implicit times of the first-order component, P1, were analysed, and the results were presented in five concentric rings and as a “sum of all groups”, see Fig. 1.

OCT measurements of the central macula of the former preterm children were performed using a SD-Cirrus HD OCT 4000 (Carl Zeiss Meditec, Dublin, CA, USA). The Macular Cube 512 × 128 was used to scan a 6 × 6 mm area with 512 horizontal A-scans and 128 vertical B-scans. The retinal thickness results are presented in three concentric circular regions: The most central region is 1 mm in diameter, the middle region is 3 mm in diameter; and the outer region is 6 mm in diameter. The circular regions were divided into nine regions according to the Early Treatment for Diabetic Retinopathy Study (ETDRS) as shown in Fig. 1.

The ETDRS map approximately corresponds with the four innermost concentric circles (14°) of the mfERG, and the central area (A1) corresponds to the central ring of the mfERG (4°) as shown in Fig. 1 [11].

### Statistical methods

SPSS 21 (IBM Corporation, Armonk, NY, USA) was used for the statistical analyses. In accordance with ISCEV recommendations, nonparametric tests were used throughout for analysis of mfERG results. The Mann-Whitey U test was used to compare the preterm group with the control group. No correction for multiple tests was performed, and *p* values should be interpreted with caution. Correlations were analysed with the Spearman correlation test. A *p* value less than 0.05 was considered statistically significant.

## Results

**Fig. 2:**
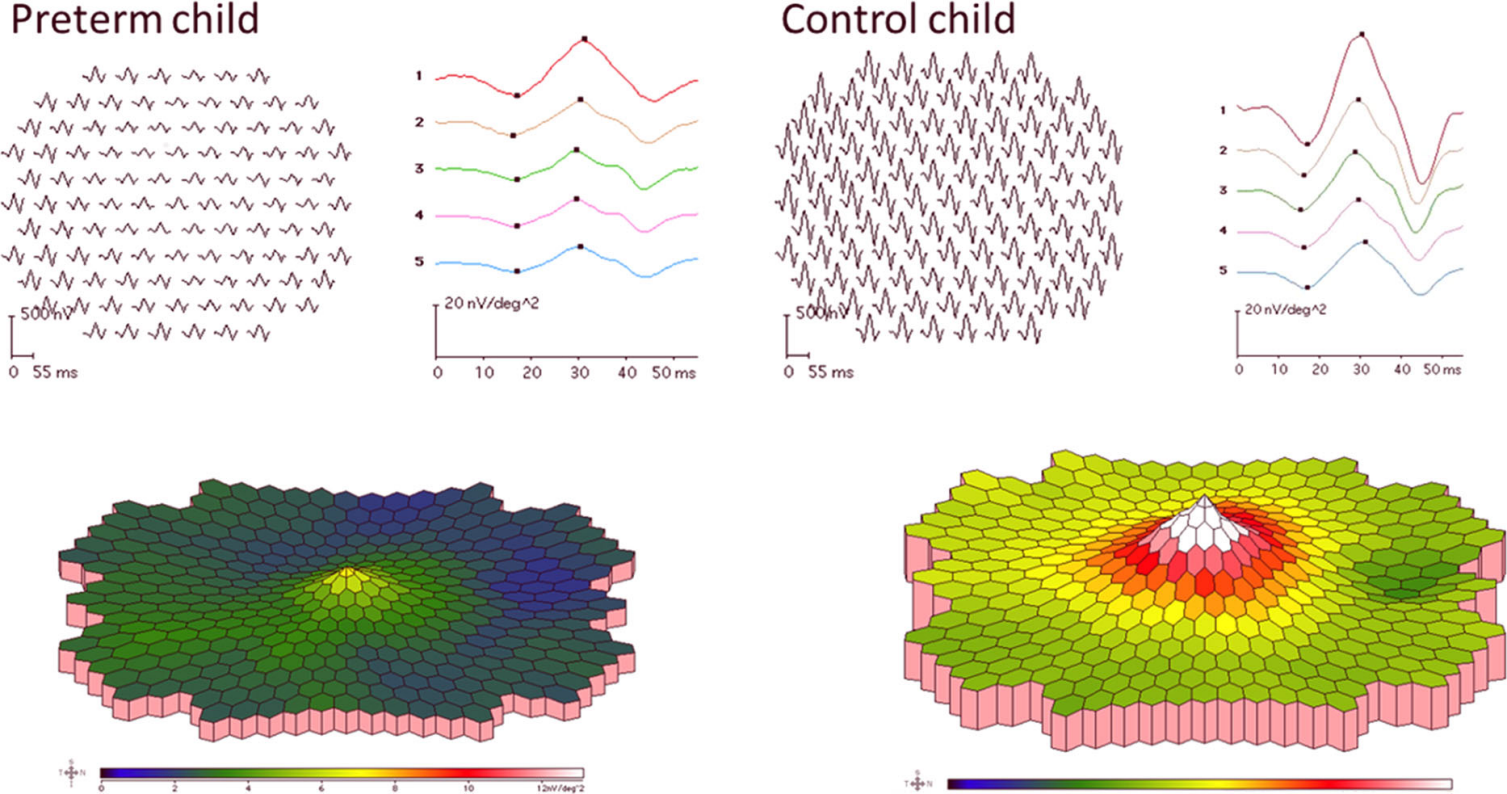
Multifocal ERG results as trace arrays and colour-coded maps, from one child in the preterm group, who had ROP grade 2 in the neonatal period, and one child in the control group

The trace array, colour map and results for Rings 1–5 as well as “Sum of groups” of mfERG from one former preterm child with ROP in the neonatal period, and one control, are shown in Fig. 2.

Median and range for amplitudes and implicit times of the P1 response in Rings 1–5 and “Sum of groups” are presented in Table 2.

**Table 2:** Implicit times and amplitudes of the multifocal ERG P1 response in Rings 1–5 and “Sum of groups” in the right eyes in the preterm group and in right or left eyes in the control group

	Preterm group *n* = 15	Control group *n* = 12	*p* value
P1 implicit time (ms) median (range)			
Ring 1	29.2 (25.0–30.8)	27.9 (25.8–30.0)	0.26
Ring 2	28.3 (24.2–30.0)	27.1 (25.0–29.2)	0.14
Ring 3	27.5 (23.3–30.0)	26.7 (25.0–28.3)	0.18
Ring 4	28.3 (24.2–29.2)	26.7 (25.0–29.2)	0.26
Ring 5	28.3 (25.0–30.0)	27.1 (25.0–30.8)	0.50
Sum of groups	28.3 (24.2–30.0)	27.1 (25.0–30.0)	0.37
P1 amplitude (nV/deg^2^) median (range)			
Ring 1	27.6 (10.1–45.5)	40.7 (21.7–51.4)	**0.01**
Ring 2	19.1 (7.6–31.4)	27.8 (16.3–35.8)	**0.02**
Ring 3	16.5 (6.3–24.2)	22.8 (12.9–29.6)	**0.02**
Ring 4	13.9 (6.0–20.0)	18.7 (11.0–26.3)	**0.01**
Ring 5	11.9 (5.5–18.5)	16.9 (10.3–25.6)	**0.01**
Sum of groups (uV)	19.2 (8.2–38.3)	24.7 (13.5–31.5)	**0.05**

Bold values indicate statistically significant p-values

**Fig. 3:**
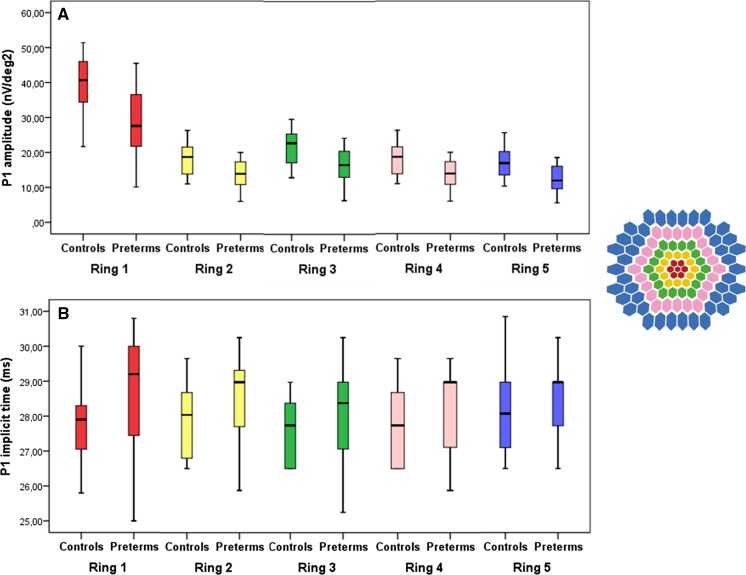
**a** Amplitudes of the P1 response in Ring 1–5 of the multifocal ERG in *right* eyes in the control group and in *right* or *left* eyes in the preterm group. **b** Implicit times of the P1 response in Ring 1–5 of the multifocal ERG in the control group and preterm group. Each *box* shows the median and interquartile range, *bars* show range

Compared to controls, P1 amplitude was significantly smaller in the preterm group. There was no significant difference in implicit times between groups (Fig. 3).

**Fig. 4:**
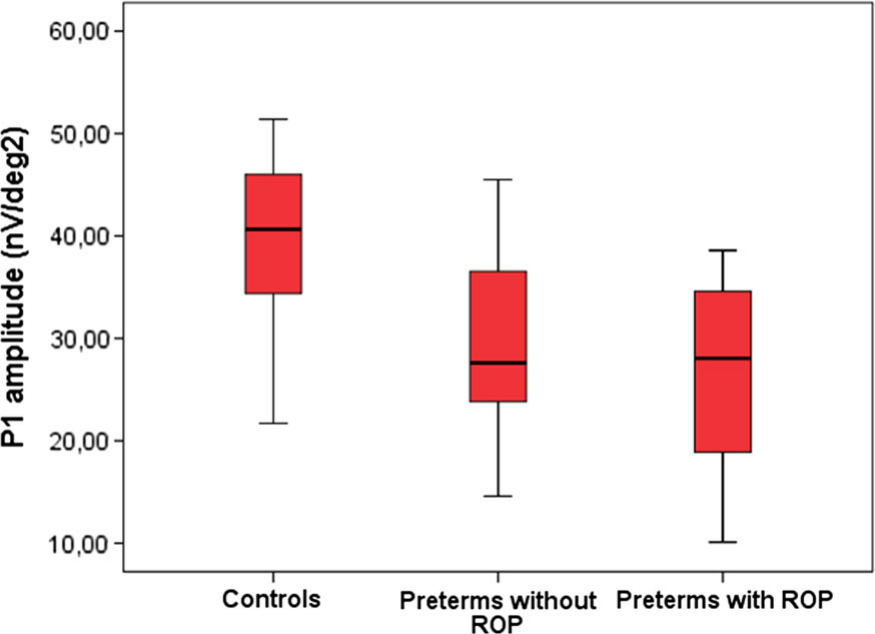
P1 amplitude of the responses in Ring 1 in multifocal ERG in *right* or *left* eyes in the controls, in *right* eyes in the preterm children without retinopathy of prematurity (ROP) and the preterm children with ROP in the neonatal period. Each *box* shows the median and interquartile range; *bars* show range

In the preterm group, there was no significant difference between children with or without ROP according to amplitudes or implicit times in any of the rings. In Fig. 4, we present the result of the P1 amplitude in Ring 1, for preterm with and without ROP and controls.

In the preterm group, OCT measurements were available in 14 of the 15 children. Median values of the central macular thickness (area A1) for the right eyes were 278 um (range 252–318). There was no significant correlation between P1 amplitude or implicit times in Ring 1 and central macular thickness. There were no significant correlations with any of the mfERG responses for age at investigation, GA, ROP or VA.

## Conclusions

Former preterm children have various visual dysfunctions, but the reason for this is not totally clear. In the present study, we have shown that the macular function measured with mfERG is reduced in prematurely born children compared to controls. Studies with mfERG in former preterm children are few, but Fulton et al. [12] have described reduced amplitudes and prolonged implicit times in preterm children with mild ROP. The amplitude reduction was greatest in the central rings and decreased in the peripheral rings, while the implicit times did not change as much with eccentricity. These results are essentially in line with the results of the present study. In the present study, we had a number of children that did not have ROP during the neonatal period. A limitation of both studies, however, is the small number of study subjects, and future studies with larger numbers of children with and without ROP in the neonatal period are needed to confirm our results.

Myopia is common in children with ROP, especially if they have been treated with laser photocoagulation or cryotherapy. Myopia is also a known reason for reduced macular function [13]. In the present study, however, most former preterm children were not myopic, and thus, the refraction cannot be an explanation for the difference between the groups.

In macular diseases such as diabetic retinopathy and macular degeneration, mfERG recordings show reduced P1 amplitudes and prolonged P1 implicit times [11, 14]. ROP is also a disease affecting the retinal vessels resulting in retinal ischaemia. It has been shown with hand-held OCT devices that preterm children with ROP can have macular cysts, oedema and subretinal fluid in the neonatal period, and although these changes resolve in time, it is not known whether the effects on macular function can persist in the future [15, 16]. Studies comparing macular morphology and macular function in older children with and without macular changes during the neonatal period could elucidate this further.

We have previously shown that central macular thickness measured with OCT is increased in former preterm children, suggesting a disturbance in the normal development of the macular region [6]. Further studies with spectral domain OCT (SD-OCT) has shown that the inner retinal cells have not completed their peripheral migration and that seems to be associated with the increased thickness [17, 18]. A recent study by Vajzovic et al. [19] using hand-held OCT showed a delayed photoreceptor development in preterm children compared to children born at term when investigated at 37 weeks of gestation and onward. It is not known whether this delay in maturation affects the photoreceptors later in life. In the present study, we could not show significant correlation between structure and function. We can only speculate that increased macular thickness in former preterm children not only is a sign of immaturity but also an indication of altered neural retinal function even if this is not confirmed by decreased visual acuity whether there is a correlation between structure and function also in healthy subjects cannot be concluded from the present study, where OCT was only performed in the children born prematurely. However, in a recent study of normal children aged 5–15 years, we also found a correlation between P1 implicit time in the inner ring of the mfERG and OCT measurements of the macular area [20].

The macular region is affected in prematurely born children regarding both morphology and function. In this small study, we could not find a correlation between macular function and visual acuity and this is probably because reduced macular function is one of the several explanations of reduced visual acuity seen in former preterm children. A larger study including children with and without ROP might give us more information about how macular function affects vision in former preterm children.
